# Traumatic pancreatic injury successfully bridged through a giant pancreatic pseudocyst

**DOI:** 10.1055/a-2739-2588

**Published:** 2025-11-21

**Authors:** Tomohisa Iwai, Masaki Nishimura, Megumi Tsukamoto, Yusuke Ozaki, Shigeru Iwase, Shin Maeda

**Affiliations:** 1 36993 Department of Gastroenterology, Fujisawa City Hospital, Kanagawa, Japan; 2 26438 Department of Gastroenterology, Yokohama City University Graduate School of Medicine, Yokohama, Japan

Pancreatic injury is a rare form of abdominal trauma and usually requires surgery for deep damage with axial deviation 1 2 . Recently, endoscopic pancreatic stenting for the disconnected pancreatic duct syndrome (DPDS) has been attempted as a minimally invasive treatment 3 4 5 , but difficult situations are often encountered. Here, we present a case of successful rendezvous stenting through a giant pancreatic pseudocyst ( Video 1 ). A 9-year-old boy fell off his bicycle and had handlebar trauma. He had a duodenal perforation and severe damage to the pancreas, resulting in DPDS ( Fig. 1 ). He first underwent mesh repair surgery for duodenal perforation, and endoscopic treatment for DPDS was unsuccessful on two attempts ( Fig. 2 ). Two weeks later, MRCP revealed a small pseudocyst, and conservative management was chosen. The patient developed postprandial abdominal pain and maintained on total parenteral nutrition (TPN) for 2 months. He was transferred to our hospital 2 months after the injury, still on TPN. MRCP performed at our hospital revealed that the pancreatic pseudocyst had enlarged to 45 mm ( Fig. 3 ). Because single-session bridging was considered impossible, two stents were placed in the pseudocyst – one via the transpapillary route and one via EUS-guided pancreatic duct drainage – to establish two points of communication between the pseudocyst and the main pancreatic duct ( Fig. 4 ). In the second session, after balloon dilation of the distal connecting part of the pancreatic duct, a guidewire inserted from the papilla of Vater was advanced across the pseudocyst into the pancreatic duct of the tail, and a transpapillary pancreatic stent was successfully placed through the pseudocyst ( Fig. 5 ). To the best of our knowledge, this is the first report of successful stent bridging through a giant pancreatic pseudocyst caused by trauma.

Traumatic pancreatic injury successfully bridged through a giant pancreatic pseudocyst.Video 1

**Fig. 1:**
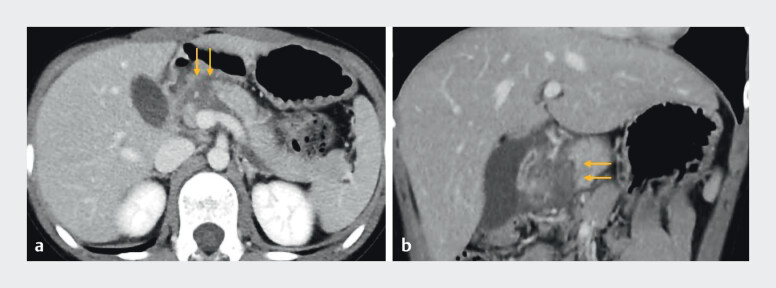
Computed tomography revealed disconnected pancreatic duct syndrome secondary to handlebar trauma (arrow). **a** Axial image. **b** Coronal image.

**Fig. 2:**
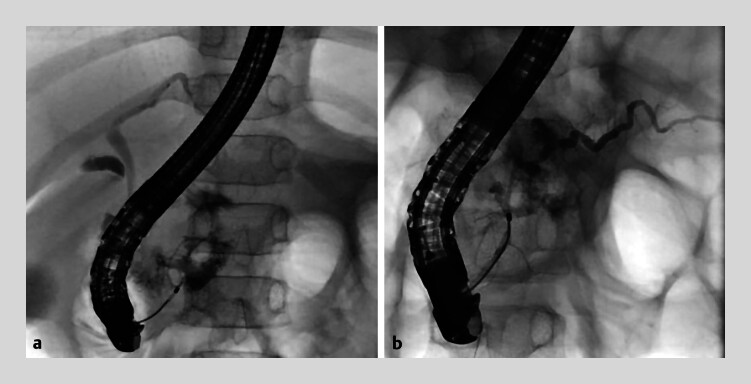
Fluoroscopic images of endoscopic retrograde cholangiography. Endoscopic pancreatic duct stenting was not achieved after two attempts. **a** First attempt, day 1 post-injury. **b** Second attempt, day 20 post-injury.

**Fig. 3:**
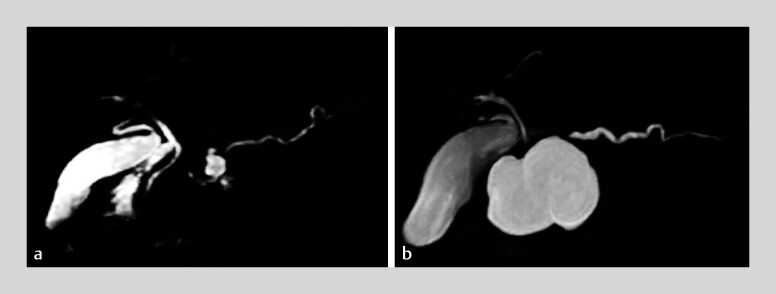
Magnetic resonance cholangiopancreatography demonstrated a pancreatic pseudocyst at the site of the trauma. **a** A small pancreatic pseudocyst was formed shortly after the injury. ( **b** ) The pseudocyst enlarged to 45 mm by the time the patient was transferred to our hospital.

**Fig. 4:**
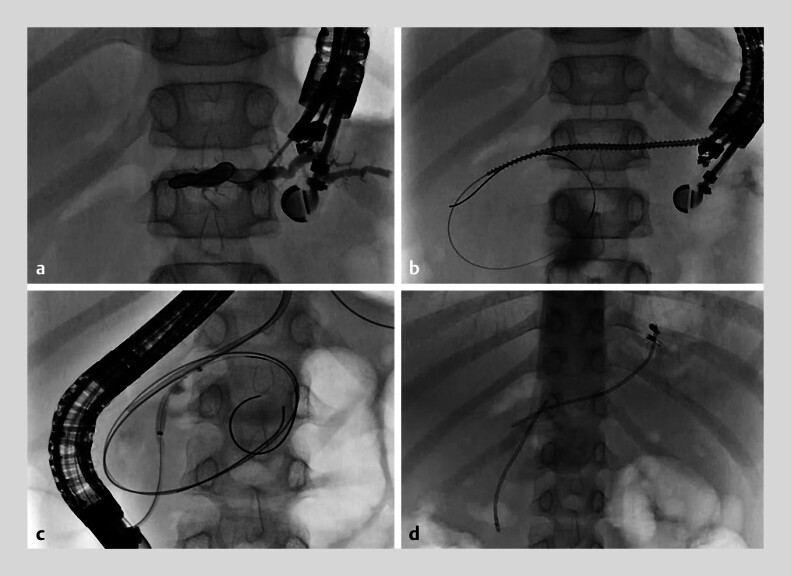
Fluoroscopic images during pancreatic stenting in the first session. **a** EUS-guided pancreatic duct drainage was performed from the distal pancreatic duct. **b** The stricture was dilated using a drill dilator. **c** From the papillary side, the stricture was dilated with a balloon dilator. **d** Two pancreatic stents were placed in the pancreatic pseudocyst.

**Fig. 5:**
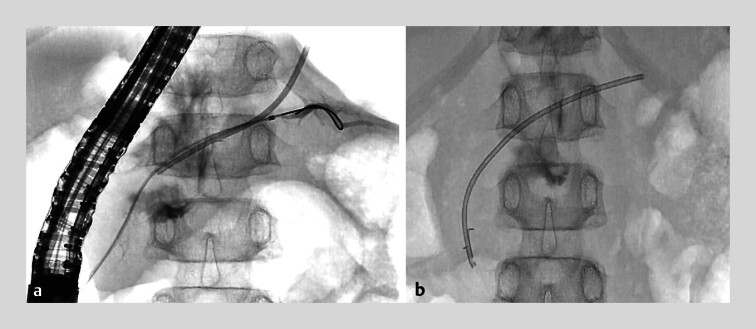
Fluoroscopic images of endoscopic retrograde cholangiography during the second session. **a** A guidewire was successfully advanced into the distal pancreatic duct through the shrunk pancreatic pseudocyst. **b** Transpapillary bridging with a single stent was achieved, and the transgastric stent was removed.

Endoscopy_UCTN_Code_TTT_1AS_2AD

## Notes

Endoscopy E-Videos https://eref.thieme.de/e-videos
*E-Videos* is an open access online section of the journal *Endoscopy* , reporting on interesting cases and new techniques in gastroenterological endoscopy. All papers include a high-quality video and are published with a Creative Commons CC-BY license. Endoscopy E-Videos qualify for HINARI discounts and waivers and eligibility is automatically checked during the submission process. We grant 100% waivers to articles whose corresponding authors are based in Group A countries and 50% waivers to those who are based in Group B countries as classified by Research4Life (see: https://www.research4life.org/access/eligibility/ ). This section has its own submission website at https://mc.manuscriptcentral.com/e-videos .
